# Multi-epitope Antigen for Specific Serological Detection of Dengue Viruses

**DOI:** 10.3390/v15091936

**Published:** 2023-09-16

**Authors:** Samuel Santos Pereira, Robert Andreata-Santos, Maria Fernanda de Castro-Amarante, Aléxia Adrianne Venceslau-Carvalho, Natiely Silva Sales, Mariângela de Oliveira Silva, Rúbens Prince dos Santos Alves, Patrícia Jungmann, Luís Carlos de Souza Ferreira

**Affiliations:** 1 Laboratory of Vaccine Development, Institute of Biomedical Sciences, Department of Microbiology, University of São Paulo, São Paulo 05508-000, Brazil; samuelbiomedicina@usp.br (S.S.P.); randreatas@gmail.com (R.A.-S.); mfamarante@gmail.com (M.F.d.C.-A.); alexiabiotec@usp.br (A.A.V.-C.); natielybiomedicina@hotmail.com (N.S.S.); angelabiotec@gmail.com (M.d.O.S.); rubens.bmc@gmail.com (R.P.d.S.A.); 2 Institut Pasteur de São Paulo, São Paulo 05508-020, Brazil; 3 General Pathology, Universidade de Pernambuco, Recife 50100-130, Brazil; patricia.jungmann@upe.br

**Keywords:** DENV, ZIKV, diagnosis, ELISA, NS1 antigen

## Abstract

Dengue is an infectious disease of global health concern that continues to require surveillance. Serological testing has been used to investigate dengue-infected patients, but specificity is affected by the co-circulation of ZIKA virus (ZIKV), which shares extensive antigen similarities. The goal of this study was the development of a specific dengue virus (DENV) IgG ELISA based on a multi-epitope NS1-based antigen for antibody detection. The multi-epitope protein (T-ΔNS1), derived from a fragment of the NS1-protein of the four DENV serotypes, was expressed in *Escherichia coli* and purified via affinity chromatography. The antigenicity and specificity were evaluated with sera of mice infected with DENV-1–4 or ZIKV or after immunization with the recombinant ΔNS1 proteins. The performance of the T-ΔNS1-based IgG ELISA was also determined with human serum samples. The results demonstrate that the DENV T-ΔNS1 was specifically recognized by the serum IgG of dengue-infected mice or humans but showed no or reduced reactivity with ZIKV-infected subjects. Based on the available set of clinical samples, the ELISA based on the DENV T-ΔNS1 achieved 77.42% sensitivity and 88.57% specificity. The results indicate that the T-ΔNS1 antigen is a promising candidate for the development of specific serological analysis.

## 1. Introduction

Dengue fever is caused by the dengue virus (DENV), i.e., the main arbovirus infecting humans [1]. DENV belongs to the Flaviviridae family and is classified into four genetically and antigenically distinct serotypes (DENV-1–4) [2] that co-circulate in tropical and subtropical areas of the world, including Brazil [3]. A total of 50 to 100 million cases of dengue infection are reported annually [4]; however, a recent study estimated 390 million dengue cases per year [4]. In Brazil, the incidence of dengue has fluctuated between 250,000 and 2 million cases annually in the twenty-first century (Pan American Health Organization; World Health Organization). Although not all notified dengue cases are serotyped, the Pan American Health Organization (PAHO) reports show that DENV serotypes 1–3 co-circulated between 2000 and 2009, and since then, all DENV serotypes have been detected in Brazil (PAHO, 2020). The geographical distribution and seasonal dominance of serotypes are heterogeneous (Brazilian Ministry of Health, 2012–2019) [5]. In addition to the four DENV serotypes, the Zika virus (ZIKV), a similar flavivirus sharing the very same epidemiologic niche, has co-circulated in Brazil since its outbreak in 2015 [6]. Considering the global impact caused by DENV infections as well as the possible occurrence of disease enhancement after DENV or ZIKV secondary infection [7], the determination of serological prevalence by means of specific surveillance methods capable of differentiating the two viruses in endemic regions is still a priority.

Mild DENV and ZIKV infections present with unspecific similar symptoms such as fever, muscle and joint pain, headache, and cutaneous rash [8]. As such, a clinical suspicion of arbovirosis alone may lead to an inaccurate diagnosis, resulting in under-reported cases [9]. In this context, serological tests are crucial laboratory tools for surveillance, having a great impact on epidemiological and public health direct actions and policies. Enzyme-linked Immunosorbent Assay (ELISA) is one of the primary serological tests used to detect anti-DENV antibodies [9]. However, commercially available tests lack the necessary specificity owing to cross-reactivity with antibodies produced against other flaviviruses, notably ZIKV [3]. Previous studies have reported difficulties combining the sensitivity and specificity of DENV ELISA tests [10]. These tests are usually performed using the Envelope (E) protein or whole virus particles from all DENV serotypes [11], which leads to extensive cross-reactivity owing to the high antigenic similarity between DENV and ZIKV [12]. Furthermore, producing antigens from each of all DENV serotypes is particularly expensive and time-consuming, making it challenging for the development of accessible serological tests. To overcome such issues, AnandaRao et al. (2005) demonstrated that multi-epitope antigens could be used as solid-phase-bound antigens in DENV ELISA to improve diagnostic performance [13]. The multi-epitope strategy avoids using the whole viral antigen, allows high epitope density, and circumvents conserved regions [13], thereby importantly improving sensitivity and specificity.

To find better-suited antigens for use as a solid phase in ELISA, our group previously reported the use of the ZIKV-NS1-derived antigen (ΔNS1) for specific antibody detection in serological tests [14]. Our strategy showed that instead of the cross-reactivity observed with the whole NS1 protein in ELISA, the ΔNS1 antigen allowed the specific detection of ZIKV and DENV antibodies. The same strategy proved successful when applied to DENV, resulting in four ΔNS1 proteins derived from each DENV serotype, which showed 82% sensitivity and 93% specificity [15]. In the present study, we aimed to produce a chimeric DENV NS1 multi-epitope recombinant antigen (T-ΔNS1) based on the fusion of the C-terminal sequences of NS1 proteins from four DENV serotypes and validate its performance as a solid-phase bound antigen in ELISA tests. The T-ΔNS1 antigen was highly expressed in bacterial cells and isolated using a two-step purification protocol. T-ΔNS1 protein was detected by antibodies against all four DENV serotypes specifically and avoided cross-reactivity with anti-ZIKV NS1 antibodies. These results reinforce the applicability of this new multi-epitope antigen in the development of specific DENV serological tests for use in regions endemic to both DENV and ZIKV.

## 2. Materials and Methods

### 2.1. Ethics Statement

All procedures involving laboratory animals were performed in accordance with the Ethical Principles of Animal Experimentation (Brazilian College of the Animal Experimentation) and approved by the Institutional Animal Care and Use Committee of the University of São Paulo (CEUA protocol number 3598020719). All human serum samples tested in this study were obtained after written informed consent. This study was approved by the Human Research Ethics Committee (CEPSH) of the Institute of Biomedical Sciences at the University of São Paulo under project numbers 5.064.047 and 36.2021.

### 2.2. T-ΔNS1 Construction and Structural Prediction

The chimeric recombinant T-ΔNS1 antigen was designed based on the fusion of the last 100 amino acids from the C-terminal of the DENV-1–4 NS1 proteins [accession numbers AHF50491 (DENV-1), CAA78918 (DENV-2), AFN80339 (DENV-3), and AEX09561 (DENV-4)], according to previous results obtained with the ZIKV ΔNS1 protein (patent application PCT number WO 2017/197477) previously published. The ZIKV ΔNS1 protein also corresponds to the 100 last amino acids of the NS1 protein. The gene encoding the DENV ΔNS1 fragments was obtained commercially (GenScript, Piscataway, NJ, USA) after codon usage optimization, subcloned in a pET-28a plasmid expression vector (Novagen), and fused in tandem using a glycine linker (6x G) and to a 6x HisTag in the N-terminal region for protein purification. Structural predictions were made using the ITASSER server (job ID S677105) [16,17]. Graphic representation was performed using PyMOL Molecular Graphics System, version 2.5.2, Schrödinger, LLC (New York, NY, USA) [18].

### 2.3. Expression and Purification of the Recombinant Chimeric T-ΔNS1

The T-ΔNS1 protein was expressed in *E. coli* ArticExpress (DE3) strain (Agilent Technologies, Saint Clare, CA, USA). Chemically competent bacteria were transformed with 100 ng of the plasmid pET-28a-T-ΔNS1 in Luria Bertani medium containing 50 µg/mL kanamycin and 20 µg/mL gentamicin at 37 °C/200 rpm, overnight. Bacterial cultures were grown using a Terrific Broth medium containing 50 µg/mL kanamycin at 37 °C/200 rpm until an optical density of 2 (O.D. = 2) was reached. For the induction of protein expression, cells were cultivated in the presence of 0.5 mM Isopropyl beta-D-thiogalactoside (IPTG) (Sigma-Aldrich, St. Louis, MO, USA) at 18 °C/200 rpm, overnight. After induction, the cells were harvested, resuspended in lysis buffer (100 mM of Tris, 500 of mM NaCl, and pH 8.5), and lysed using a cell homogenizer APLAB-10 (Artepeças, São Paulo, SP, Brazil). Protein fractions were separated using centrifugation, and the insoluble protein fraction was recovered and solubilized in denaturation buffer (100 mM of Tris, 500 mM of NaCl, 8 M of Urea, and pH 8.5). The solubilized T-ΔNS1 was refolded in 2 L of the buffer containing 100 mM of Tris, 500 mM of NaCl, 10% glycerol, and pH 8.5 using pulsatile dilution [19]. The refolded protein was centrifuged and filtered using a 0.22 µM nitrocellulose membrane (Sartorius, Gottingen, Germany). Protein purification was performed via nickel affinity chromatography using a 5 mL HisTrap HP column (GE Healthcare Life Sciences, Chicago, IL, USA) previously equilibrated with a buffer (100 mM of Tris, 500 mM of NaCl, 10% glycerol, and pH 8.5). A crescent gradient with a buffer containing imidazole (100 mM of Tris, 500 mM of NaCl, 1 M of Imidazole, 10% glycerol, and pH 8.5) was used for elution. The purified protein was analyzed using SDS-PAGE (polyacrylamide gel 15%) under denaturing conditions and using Western blot with an anti-HisTag monoclonal antibody diluted 1:3000 (Sigma-Aldrich, St. Louis, MO, USA), detected with goat anti-IgG mouse (Sigma-Aldrich, St. Louis, MO, USA) and diluted 1:4000. The ECL Select Western Blotting Detection Reagent (GE Healthcare Life Sciences, GE Healthcare Life Sciences, Chicago, IL, USA) was used for detection, according to a protocol previously described [20]. The nitrocellulose membranes were visualized in ChemiDoc system (BIO-RAD Laboratories, Inc).

### 2.4. Production of Anti-ΔNS1, Anti-T-ΔNS1, Anti-DENV-1–4, and Anti-ZIKV Polyclonal Mouse Hyperimmune Sera

DENV-1–4 anti-ΔNS1 and anti-T-ΔNS1 polyclonal production of antibodies were induced in 6- to 8-weeks-old BALB/c mice (4 animals/group), which were inoculated with 10 µg of DENV-1–4 ΔNS1 or T-ΔNS1 adjuvanted with 1 µg of Heat-Labile Toxin 1 (LT-1) or with 25 µg of Alum (Brenntag), respectively. Animals were subjected to an immunization regimen of three doses on days 0, 14, and 28 via the subcutaneous route. Blood samples were collected before immunization and 14 days after each dose to obtain serum. ZIKV anti-ΔNS1 polyclonal antibodies were previously obtained following immunization with ZIKV ΔNS1 protein. To make anti-DENV and anti-ZIKV hyperimmune serum, immunodeficient AG129 mice (5 animals/group) were experimentally infected via a subcutaneous route, into the footpad, with nonlethal doses of DENV-1–4 or ZIKV (GenBank access numbers: JX669467, M29095, KC425219, GU289913.1, and KU729217). Animals inoculated with DENVs 1, 3, and 4 received 10^4^ PFU (Particle-forming Unit), and animals inoculated with DENV 2 and ZIKV received 50 and 10 PFU, respectively. A second dose was administrated in the animals inoculated with DENVs 1, 2, and 4. In contrast, negative sera were produced after inoculating the same volume of Dulbecco’s modified Eagle medium (DMEM, Life Technologies, Carlsbad, CA, USA).

### 2.5. Immunological Detection and Performance of T-ΔNS1

Immunological detection of anti-ΔNS1, anti-T-ΔNS1, anti-DENV, and anti-ZIKV mouse hyperimmune sera was performed using ELISA. For the detection of DENV ΔNS1 fragments on T-ΔNS1, anti-ΔNS1-specific hyperimmune sera were used. ELISA plates (Corning Incorporated, Corning, NY, USA) were coated with 100 µL of solutions containing 2 ng/µL of DENV-1–4 ΔNS1 and 8 ng/µL of T-ΔNS1 diluted in carbonate buffer (32.5 mM of NaHCO, 14.9 mM of Na2CO3, and pH 9.6). Plates were blocked with 3% skimmed milk (Molico, Nestlé) and 0.5% bovine serum albumin (INLAB) diluted in Phosphate-buffered Saline Tween 0.05% (PBS-T) at 37 °C for 2 h, washed thrice with PBS-T, and incubated with DENV-1–4 anti-ΔNS1 (*n* = 4) or anti-T-ΔNS1 (*n* = 4) mouse hyperimmune serum diluted 1:500 and ZIKV anti-ΔNS1 (*n* = 7) diluted 1:100 in block solution at 37 °C for 1 h. The plates were washed thrice with PBS-T and incubated with anti-IgG mouse conjugated to horseradish peroxidase (HRP) (Sigma-Aldrich, St. Louis, MO, USA) diluted 1:4000 in a block solution. The plates were rewashed and 100 µL of o-phenylenediamine dihydrochloride (OPD, Sigma-Aldrich) with H_2_O_2_ added for color development at room temperature for 15 min. The reaction was stopped with 50 µL of 2N H_2_SO_4_, and the absorbance was measured at 492 nm using a plate reader (BioTek, Agilent Technologies, Saint Clare, CA, USA). Alternatively, plates were coated with 100 µL of 2 ng/µL of DENV-1–4 ΔNS1 and 8 ng/µL of T-ΔNS1 to react with anti-DENV (*n* = 5) or anti-ZIKV (*n* = 5) mouse hyperimmune serum diluted 1:100 in block solution. Subsequently, the same procedure described above was followed.

Additional tests, aimed at evaluating the recognition of conformational and linear epitopes using antibodies produced in mice after immunization, were performed using ELISA with plates coated with 100 µL of 8 ng/µL of heated-denatured and non-denatured T-ΔNS1 protein. For denaturation, T-ΔNS1 was heated at 100 °C for 10 min and then incubated on ice for 15 min. ELISA was performed as previously described using anti-DENV-1–4 mouse hyperimmune serum (*n* = 5) diluted 1:100 in the block solution. To perform the T-ΔNS1-based DENV IgG ELISA, plates were coated with 100 µL of 8 ng/µL of the T-ΔNS1, in order to achieve 2oo ng of each ΔNS1 antigen per well. The tested serum samples were pre-incubated with 2.2 µg of the ZIKV ΔNS1 recombinant protein and diluted 1:100 in block solution. The T-ΔNS1-based DENV IgG ELISA performance was evaluated using 101 human serum samples. DENV-positive (*n* = 32) and -negative (*n* = 69) samples were classified according to the reactivity obtained after evaluation using the ΔNS1-based DENV IgG ELISA (cutoff 0.5) previously published by our group [15].

### 2.6. Statistical Analyses

Statistical determination of sensitivity and specificity was performed using MedCalc software version 20.111. Additional graphs and statistical analyses were performed using GraphPad Prism version 9.0. Differences were considered statistically significant at *p* < 0.05.

## 3. Results

### 3.1. The T-ΔNS1 Antigen Displays Conformational Epitopes of All DENV Serotypes

**Figure 1:**
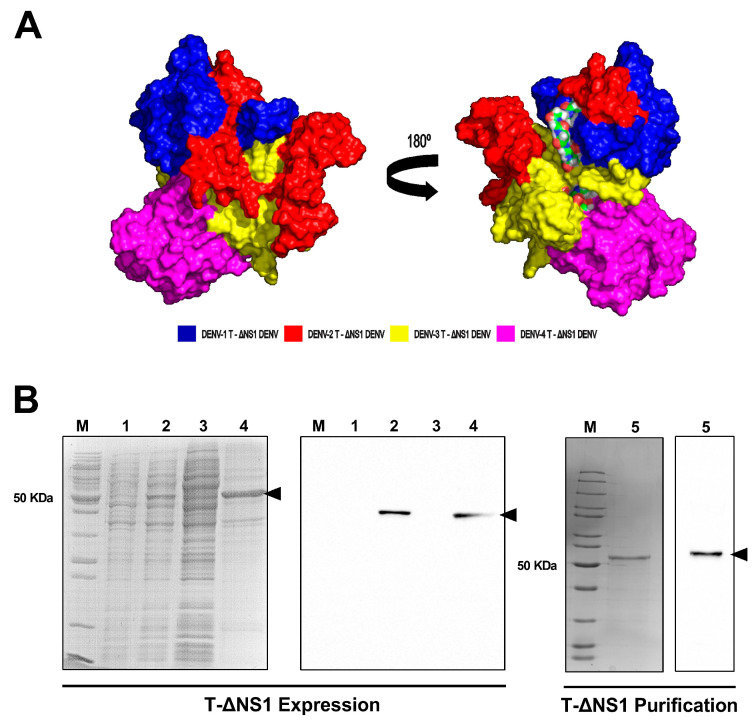
Structural prediction, expression, and purification of the T-ΔNS1 recombinant protein. (**A**) Three-dimensional structural model of T-ΔNS1 surface structure, where colors indicate DENV-1–4 ΔNS1 fragments: blue (DENV-1), red (DENV-2), yellow (DENV-3), and pink (DENV-4). (**B**) Expression analyses: Coomassie blue-stained 15% polyacrylamide SDS-PAGE gel (left) and Western blot analysis with anti-HisTag monoclonal antibody (right): M (molecular weight marker), 1 (whole-cell extract of a non-induced culture), 2 (whole-cell extract of induced culture), 3 (soluble protein fraction), and 4 (insoluble protein fraction). Purification analyses: Coomassie blue-stained 15% polyacrylamide gel (left) and Western blot (right) showing the purified T-ΔNS1 protein (5).

Chimeric recombinant T-ΔNS1 was obtained by fusion of the DENV-1–4 NS1 proteins C-terminal regions (ΔNS1). To provide proper stability and a core for appropriate protein folding, linkers composed of six glycine residues were inserted between each ΔNS1 sequence. Structural prediction showed four distinguished sections with glycine bonds and DENV-3 ΔNS1 acting as T-ΔNS1 core regions (Figure 1A). In silico analyses indicated that all DENV serotype fragments were present on T-ΔNS1. However, DENV-2 and DENV-3 were partially buried in the structure and may be partially hidden for antibody binding. Notwithstanding, T-ΔNS1 may have accessible exposed NS1 epitopes for all DENV serotypes and could detect DENV-1–4 anti-NS1 antibodies. The T-ΔNS1 protein was expressed by the *E. coli* Arctic Express strain with a molecular weight of approximately 50 kDa, recovered from the insoluble fraction of the cellular extract (Figure 1B), refolded, and purified via affinity chromatography with a yield of 1.55 mg/L. SDS-PAGE and Western blot analyses showed >90% purity (Figure 1B). These results indicate that the T-ΔNS1 multi-epitope recombinant protein was highly expressed and properly purified to be used as a solid-phase antigen in ELISA assays.

### 3.2. The T-ΔNS1 Is Immunogenic against DENV-1–4 Hyperimmune Mice Serum

**Figure 2:**
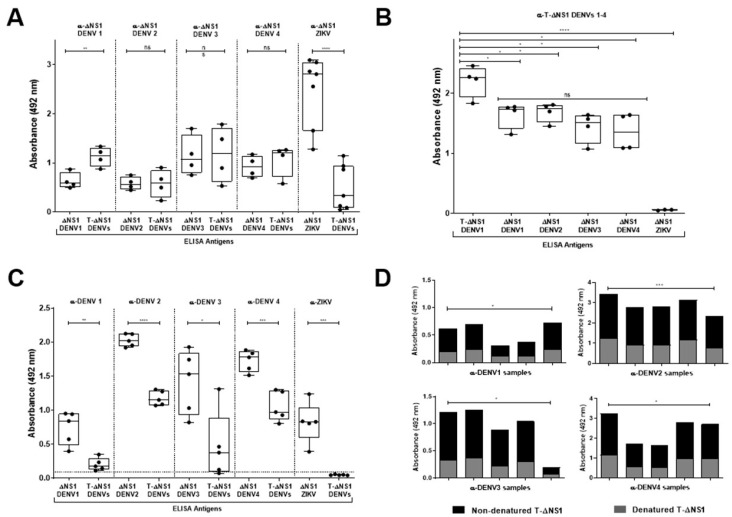
Evaluation of the T-ΔNS1 immunological features. (**A**) Comparative reactivity among T-ΔNS1, DENV-1–4 ΔNS1, and ZIKV ΔNS1 proteins with anti-ΔNS1 from mice previously immunized with ΔNS1 DENV-1–4 or ΔNS1 ZIKV. Statistical significance was calculated using a *t*-test (** *p* < 0.008, **** *p* < 0.0001, and ns = non-significant). (**B**) Reactivity of DENV-1–4 ΔNS1 and ZIKV ΔNS1 proteins with anti-T-ΔNS1 from mice previously immunized with T-ΔNS1. Statistical significance was determined using a *t*-test (* *p* < 0.01, ** *p* < 0.006, **** *p* < 0.0001, and ns = non-significant). (**C**) Comparative reactivity between T-ΔNS1 and DENV-1–4 ΔNS1 with anti-DENV-1–4 and anti-ZIKV from mice experimentally infected with live virus particles. Statistical significance was calculated using a *t*-test (* *p* < 0.01, ** *p* < 0.001, *** *p* < 0.0005, and **** *p* < 0.0001). The dotted line indicates two times the mean of reactivity of DENV-negative samples. (**D**) Reactivity of anti-DENV-1–4 mouse hyperimmune serum with conformational epitopes on non-denatured T-ΔNS1 protein and linear epitopes on denatured T-ΔNS1 protein. Statistical significance was calculated using a *t*-test (* *p* < 0.03 and *** *p* < 0.0001).

To confirm that the chimeric protein exposes the epitopes of all DENV serotypes, the first T-ΔNS1 immunological characterization as a solid-phase antigen in ELISA was performed with T-ΔNS1 detection using mouse hyperimmune sera. The T-ΔNS1 and DENV-1–4 or ZIKV ΔNS1 were exposed to anti-ΔNS1 polyclonal mouse serum obtained after immunization with DENV-1–4 or ZIKV ΔNS1. Similar reactivity was observed with T-ΔNS1 and DENV-2–4 ΔNS1, while statistically higher detection was observed for T-ΔNS1 in comparison with DENV-1 ΔNS1 (Figure 2A). Moreover, limited cross-reactivity was observed with anti-ΔNS1 ZIKV and T-ΔNS1. On the other hand, while similar detection of all DENV-1–4 ΔNS1 proteins by antibodies produced after mice immunization with T-ΔNS1 was observed (Figure 2B), lower detection levels of T-ΔNS1 compared to the individual DENV-1–4 ΔNS1 proteins were monitored by introduction of anti-NS1 antibodies produced after DENV-1–4 infections in AG129 mice, especially among anti-DENV-1 and anti-DENV-3 (Figure 2C), as suggested by structural prediction (Figure 1A). However, the detection levels were still far above the cutoff values (Figure 2C). Under such conditions, a lack of cross-reactivity against anti-ZIKV NS1 antibodies was observed for the solid-phase T-ΔNS1 protein (Figure 2C). Furthermore, T-ΔNS1 reactivity seemed to rely on conformational epitopes, as ELISA reactivity was significantly reduced after protein denaturation (Figure 2D). Collectively, these results indicate that the T-ΔNS1 preserves the immunogenicity of all DENV ΔNS1 proteins and reduces cross-reactivity against anti-ZIKV antibodies.

### 3.3. The T-ΔNS1 Antigen Specifically Detects Previous DENV-1–4 Infections

**Figure 3:**
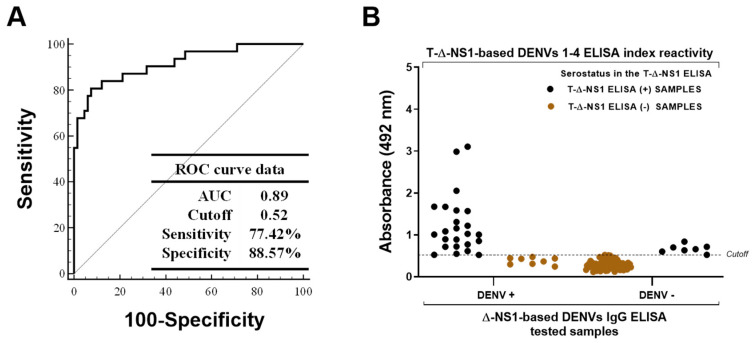
Performance of the T-ΔNS1-based DENV IgG ELISA. (**A**) Receiver operating characteristic (ROC Curve) analysis of the T-ΔNS1-based DENV IgG ELISA index reactivity. The parameters determined with the ROC curve were as follows: area under the curve (AUC) of 0.89 and sensitivity and specificity of 77.42% and 88.57%, respectively. (**B**) Reactivity index of DENV (+) and DENV (−) human serum samples tested with the T-ΔNS1-based ELISA. The dotted line indicates the cutoff value (0.52). Black and brown dots represent positive and negative samples in the T-ΔNS1-based ELISA, respectively.

We used a human serum sample panel to evaluate the T-ΔNS1-based DENV IgG ELISA for detecting specific DENV antibodies (*n* = 101). All samples were tested, and the index reactivity was used to determine the receiver operating characteristic (ROC). When the cutoff was established at 0.52, the test achieved high accuracy, as indicated by the Area Under the Curve (AUC) value of 0.89, with a sensitivity and specificity of 77.42% and 88.57%, respectively (Figure 3A). In these conditions, 24 of 32 DENV-positive samples were positive in the T-ΔNS1-based DENV IgG ELISA (Figure 3B and Table 1), while 6 of the 8 DENV-positive samples, which were found negative, stayed borderline to the cutoff, with O.D. values ranging between 0.31–0.47. Moreover, seven of these eight negative samples showed low reactivity in the ΔNS1-based DENV IgG ELISA, with O.D. values lower than 0.9 (Table S1). Conversely, only 7 of the 69 DENV-negative samples were positive in the T-ΔNS1-based DENV IgG ELISA (Figure 3B and Table S1). Similarly, the reactivity of these DENV-negative samples remained borderline within the cut-off value. Together, these results demonstrate the performance of the T-ΔNS1 constructs as a target antigen for developing valuable serological tests to detect previous DENV infections specifically.

## 4. Discussion

Commercial dengue ELISA kits show limited and variable performance, particularly in areas where ZIKV is also endemic [3,10]. Therefore, advances are required to improve the accuracy of serological tests, including the discovery of new antigens as tools for detecting dengue-specific antibodies. This study showed a multiepitope recombinant protein based on the fusion of C-terminal NS1 fragments from DENV-1–4, which could detect specific anti-NS1 IgG against all DENV serotypes. The multi-epitope T-ΔNS1 recombinant protein produced in *E. coli* was successfully recognized using anti-ΔNS1 antibodies against the four DENV serotypes. Moreover, anti-T-ΔNS1 polyclonal serum reacted with DENV-1–4 ΔNS1 recombinant proteins, which corroborated both the immunogenicity and antigenicity of this new chimeric antigen. T-ΔNS1 was recognized by serum from DENV-1–4 infected mice but not by serum from ZIKV-infected animals, which confirmed the specific detection of antibodies generated after the induced infections. Finally, when the T-ΔNS1 antigen was used as a solid-phase-bound antigen in IgG ELISA against patients’ sera that were positive and negative for DENV infection, 77.42% sensitivity and 88.57% specificity were observed. Altogether, these results indicate that T-ΔNS1 preserved the immunological determinants of native DENV proteins and efficiently detected anti-NS1 DENV IgG with reduced recognition using ZIKV antibodies.

The T-ΔNS1 multi-epitope protein was constructed with the four ΔNS1 DENV serotypes and is expected to detect antibodies produced during the primary infection, which is an advantage on serological diagnostics based on one DENV serotype antigen. The T-ΔNS1 construction derived from previous observations based on the C-terminal fragment NS1 (ΔNS1) from ZIKA [14] and DENV [15]. We showed that the ΔNS1 antigens allow recognition with anti-ZIKV or anti-DENV antibodies with limited cross-reactivity. This peculiar feature of the ΔNS1 antigens may be explained by the fact that the C-terminal region of the NS1 protein contains strain-specific B-cell epitopes capable of differentiating anti-ZIKV from anti-DENV antibodies. Moreover, the observed specificity could be related to electrostatic differences between the C-terminal NS1 of flaviviruses [21]. Indeed, previous pieces of evidence have demonstrated the low cross-reactivity of anti-NS1 antibodies raised in patients infected with different arboviruses [22], with the exception of DENV and ZIKV.

DENV-NS1-based immunoassays usually allow antibody detection even during the acute phase of infection [23]. Nonetheless, the conserved sequences shared by DENV and ZIKV NS1 result in high cross-reactivity of antibodies raised in infected patients. A demonstration that an antigen based on C-terminal NS1 epitopes could confer higher specificity to antibody detection represented an important step toward the development of improved serological tests capable of differentiating ZIKV and DENV infections [14,24]. The development of a recombinant chimeric DENV NS1 antigen preserved the high specific serological detection based on the use of a single antigen and, therefore, avoided the demand to generate four antigens of each DENV serotype. Ananda Rao et al. (2005) previously reported a multi-epitope-based immunoassay based on DENV NS1. Nonetheless, the recombinant antigen was designed to encompass four linear immunodominant epitopes, corresponding to 15 amino acid residues located at the N-terminal end of NS1 [25]. According to the authors, this region contains IgM-specific epitopes and, thus, could be useful for the detection of this type of immunoglobulin. In contrast, T-ΔNS1 shows unique features associated with conformational epitopes on the C-terminal end of the protein and allows the detection of IgG antibodies.

The T-ΔNS1 structure comprises glycine linkers inserted between each DENV ΔNS1 fragment to provide stability and flexibility to the recombinant chimeric antigen. The glycine linker is widely used to design multi-epitope proteins [13,26], which has been demonstrated for DENV/ZIKV (hexa- or tetra-glycine linker) and Leptospira antigens (tetra-glycine linker) [27,28]. The role of glycine linkers in the T-ΔNS1 composition can be observed in Figure 1A, as they support the DENV-3 ΔNS1 fragment to form the T-ΔNS1 core and, consecutively, the whole T-ΔNS1 folding. In silico structural analyses were performed to investigate whether DENV-1–4 ΔNS1 epitopes were exposed and accessible to the newly designed T-ΔNS1 antigen. The results demonstrated that ΔNS1 fragments from all DENV serotypes were exposed and likely accessible for antibody recognition, except for DENV-3 ΔNS1, which was partially buried in the recombinant protein. Immunological assays with anti-ΔNS1, anti-T-ΔNS1, and anti-DENV hyperimmune serum confirmed that all DENV fragments were accessible to T-ΔNS1. However, lower reactivity was observed with serum anti-DENV-1 and anti-DENV-3, which were partially hidden in the predicted T-ΔNS1 structure. In addition, we demonstrated that T-ΔNS1 encompasses such particular conformational epitopes, as a loss of reactivity was then observed with heat-denatured T-ΔNS1. Our previous observations showed that most specific antibodies detected by the ZIKV ΔNS1 protein reacted with conformational epitopes [14]. Thus, the present T-ΔNS1 antigen supports a viable alternative to specifically detect anti-DENV-1–4 antibodies, particularly in regions where the surveillance of all DENV serotypes is missed.

As the first implementation of the T-ΔNS1 as a solid-phase antigen to detect anti-DENV antibodies, we evaluated a human serum sample panel, and ΔNS1-based DENV IgG ELISA was previously tested. In this study, the T-ΔNS1-based DENV IgG ELISA achieved sensitivity and specificity values of 77.42% and 88.57%, respectively. Our results were superior or equivalent to those of commercially available tests previously reported in the literature, including the Panbio Dengue virus IgG Capture ELISA (56% sensitivity and 95% specificity) and the SD Bioline Dengue IgG ELISA (89% sensitivity and 64% specificity) [10]. Regarding experimental tests, we achieved comparable performance to tests based on full-length DENV NS1 proteins, such as the DENV-1–4 NS1 IgG ELISA (83–87% sensitivity, 81–93% specificity) [29], the DENV2 NS1 IgG ELISA (83% sensitivity and 88% specificity) [30]. Although envelope-based tests showed greater specificity than the T-ΔNS1-based DENV IgG ELISA [31], studies conducted before the ZIKV outbreak did not encounter cross-reactivity issues between DENV and ZIKV. Therefore, our data strongly suggest that T-ΔNS1 has the potential to be used as a DENV-specific pan antigen in serological tests aimed at efficiently detecting previous infections with DENV, even in ZIKV-endemic regions.

In conclusion, this study introduces a new multi-epitope antigen, named T-ΔNS1, derived from the DENV-1–4 C-terminal NS1 protein, previously reported to confer specificity in the detecting of DENV- and ZIKV-specific antibodies. The T-ΔNS1 showed a relatively good performance using an in-house ELISA protocol and, hence, can be considered a promising alternative for developing future serological tests, particularly for dengue serological surveillance studies in ZIKV endemic countries.

## Figures and Tables

**Table 1:** Analyses of the T-ΔNS1-based DENV IgG ELISA performance.

	T-ΔNS1-Based DENV IgG ELISA	
	Positive	Negative
**DENV (+)** **Samples (*n* = 32)**	24	8
**DENV (−)** **Samples (*n* = 69)**	7	62
**Sensitivity**	77.42%	
**Specificity**	88.57%	

## Data Availability

The data presented in this study are available upon request from the corresponding author.

## Supplementary Materials

The following supporting information can be downloaded at https://www.mdpi.com/article/10.3390/v15091936/s1, Table S1: absorbance in the T-ΔNS1-based DENV IgG and ΔNS1-based DENV IgG ELISAs.
