# Supplementary material for: Multi-epitope Antigen for Specific Serological Detection of Dengue Viruses
Source: Viruses. 2023 Sep 16;15(9):1936. doi: 10.3390/v15091936 (PMC10535193; doi:10.3390/v15091936)
Supplement: Supplementary file 1 [file viruses-15-01936-s001.zip › viruses-2585315-supplementary.pdf]

Supplementary Material

Multi-epitope Antigen for Specific Serological Detection of Dengue Viruses

Samuel Santos Pereira, Robert Andreata Santos, Maria Fernanda de Castro-Amarante, Aléxia Adrianne Venceslau-Carvalho, Natiely Silva Sales, Mariângela de Oliveira Silva, Rúbens Prince dos Santos Alves, Patrícia Jungmann, Luís Carlos de Souza Ferreira*

*** Correspondence:** lcsf@usp.br

1 Supplementary table

TABLE S1. Absorbance in the T-ΔNS1-based DENV IgG and ΔNS1-based DENV IgG ELISAs

**Sample ID***	**ELISA test**		**Sample ID**	**ELISA test**	
	**ΔNS1-based**	**T-ΔNS1-based**		**ΔNS1-based**	**T-ΔNS1-based**
**01**	2,6795	1,5835	**52**	0,09	0,194
**02**	0,9115	0,4745	**53**	0,285	0,3385
**03**	1,0085	0,8885	**54**	0,193	0,6025
**04**	0,924	0,524	**55**	0,243	0,378
**05**	0,512	0,3105	**56**	0,2045	0,513
**06**	1,563	1,3095	**57**	0,155	0,429
**07**	1,606	0,772	**58**	0,1365	0,4525
**08**	1,3345	0,8525	**59**	0,1035	0,3255
**09**	3,312	1,569	**60**	0,225	0,256
**10**	0,961	1,6735	**61**	0,1595	0,3115
**11**	2,0445	1,0105	**62**	0,103	0,469
**12**	1,811	1,0055	**63**	0,1735	0,282
**13**	2,507	1,67	**64**	0,219	0,453
**14**	3,781	2,0525	**65**	0,167	0,5205
**15**	0,6015	0,2435	**66**	0,164	0,66
**16**	1,059	0,299	**67**	0,2585	0,47
**17**	3,164	1,0835	**68**	0,1005	0,143
**18**	4	3,106	**69**	0,135	0,227
**19**	4	2,986	**70**	0,1565	0,1885
**20**	1,7725	0,445	**71**	0,3165	0,2875
**21**	0,5035	0,549	**72**	0,1235	0,141
**22**	2,506	1,0255	**73**	0,148	0,5055
**23**	0,5075	0,523	**74**	0,197	0,2965
**24**	2,5085	1,156	**75**	0,1165	0,1325
**25**	1,5945	0,616	**76**	0,1545	0,2515
**26**	0,9115	0,716	**77**	0,095	0,156
**27**	0,7065	0,437	**78**	0,219	0,3755
**28**	0,618	0,4295	**79**	0,151	0,372

**29**	1,823	0,8965	**80**	0,2375	0,3015
**30**	0,704	0,365	**81**	0,2255	0,3085
**31**	1,091	0,7225	**82**	0,2505	0,348
**32**	2,8175	1,218	**83**	0,1885	0,307
**33**	0,1495	0,1225	**84**	0,085	0,114
**34**	0,1175	0,259	**85**	0,199	0,3345
**35**	0,1155	0,229	**86**	0,3675	0,7015
**36**	0,1325	0,229	**87**	0,2205	0,229
**37**	0,3725	0,3785	**88**	0,191	0,149
**38**	0,0865	0,184	**89**	0,109	0,2575
**39**	0,1785	0,333	**90**	0,218	0,337
**40**	0,1205	0,191	**91**	0,434	0,256
**41**	0,1675	0,257	**92**	0,1715	0,2685
**42**	0,08	0,173	**93**	0,226	0,2695
**43**	0,228	0,4535	**94**	0,1635	0,71675
**44**	0,251	0,5235	**95**	0,0665	0,206
**45**	0,186	0,4505	**96**	0,3215	0,63175
**46**	0,207	0,839	**97**	0,125	0,17775
**47**	0,167	0,2905	**98**	0,2365	0,3735
**48**	0,1465	0,405	**99**	0,1685	0,26
**49**	0,09	0,2465	**100**	0,212	0,3135
**50**	0,141	0,231	**101**	0,179	0,1655
**51**	0,201	0,161			

*Sample ID numbered from 01 to 32 and 33 to 101 indicated the original DENV positive serum and the original 69 DENV negative serum, respectively.
